# Relationship between Tilt Sensation Ability and Lower Limb Injuries in Junior Athletes

**DOI:** 10.3390/ijerph21070947

**Published:** 2024-07-19

**Authors:** Maki Tanaka, Yuka Inoue, Megumi Gonno, Teruo Nomura, Kyosuke Oku, Tomoyuki Matsui, Machiko Hiramoto, Tetsuya Miyazaki, Hitoshi Koda, Yuya Watanabe, Yoshihiro Kai, Toru Morihara, Noriyuki Kida

**Affiliations:** 1 Department of Childhood Education, Faculty of Human Development and Education, Kyoto Tachibana University, 34 Yamada-cho, Oyake, Yamashina-ku, Kyoto 607-8175, Japan; 2 Underprograms of Applied Biology, Kyoto Institute of Technology, Hashikami-cho, Matsugasaki, Sakyo-ku, Kyoto 606-8585, Japan; hamu56clear@gmail.com; 3 Department of Childhood Education, Faculty of Childhood Education, Nagoya Women’s University, 3-40 Shioji-cho, Mizuho-ku, Nagoya-shi 467-8610, Japan; gonno@nagoya-wu.ac.jp; 4 Faculty of Arts and Sciences, Kyoto Institute of Technology, Hashikami-cho, Matsugasaki, Sakyo-ku, Kyoto 606-8585, Japan; note0420@gmail.com (T.N.); kyosuke.oku@gmail.com (K.O.); kida@kit.ac.jp (N.K.); 5 Marutamachi Rehabilitation Clinic, 12 Nishinokyo Kurumazakacho Nakagyo-ku, Kyoto 604-8405, Japan; matsui.tomoyuki.sports.reha@gmail.com (T.M.); true.to.your.heart810@gmail.com (M.H.); mtsports0512@gmail.com (T.M.); toru4271@koto.kpu-m.ac.jp (T.M.); 6 Department of Rehabilitation Sciences, Faculty of Allied Health Sciences, Kansai University of Welfare Sciences, Asahigaoka 3-11-1, Kashiwara-shi 582-0026, Japan; h-koda@tamateyama.ac.jp; 7 Department of Sports Study, Faculty of Sport Study, Biwako Seikei Sport College, 1204 Kitahira, Otsu-shi 520-0503, Japan; watanabe-yuy@bss.ac.jp; 8 Department of Physical Therapy, Faculty of Health Sciences, Kyoto Tachibana University, 34 Yamada-cho, Oyake, Yamashina-ku, Kyoto 607-8175, Japan; kai-y@tachibana-u.ac.jp

**Keywords:** tilt sensation ability, proprioception, lower limb injuries, junior athletes

## Abstract

The purpose of this study was to devise a tilt sensation measurement method to evaluate ankle proprioception and to examine its reliability. It was also used to determine the relationship among tilt sensation abilities, physical development, and lower limb injuries in junior athletes. In this study, a step platform created tilt angles. Participants with eye masks answered “yes” or “no” to sensing a tilt, evaluated over nine or seven trials. Experiment 1 involved 22 university students (20.6 ± 0.9 years). The minimum angle at which a tilt could be sensed while standing on both feet was determined, and measurements were taken again to examine reliability. Experiment 2 involved 40 junior athletes (12.3 ± 2.0 years), where the minimum angle for tilt sensation was obtained, and medical checks were conducted to assess injuries in the knee, lower leg, and foot. Reliability studies showed a moderately significant correlation between the first and second sessions (*r* = 0.504, *p* = 0.017), suggesting the reliability of the experimental method. The proportion capable of sensing a tilt of 1.1° and 1.6° was significantly higher in junior high school students than in elementary school students (1.1°; *χ*^2^ = 8.839, *p* = 0.003. 1.6°; *χ*^2^ = 4.038, *p* = 0.044). The group unable to sense a tilt of 1.6° and 2.1° had a significantly higher positive rate of knee injuries compared to the sensed group among junior high school students (1.6°; *χ*^2^ = 4.622, *p* = 0.032. 2.1°; *χ*^2^ = 4.622, *p* = 0.032). Our findings suggested that a reduced tilt sensation ability was associated with knee injuries in junior high school students. Utilizing our devised tilt sensation assessment could play a crucial role in preventing and detecting early injuries in junior high school students.

## 1. Introduction

Maintaining posture under various conditions is essential in sports. Posture control requires proper motor control. Research in motor control is conducted from various perspectives, including motor learning, the coordination between nerves and muscles, and injury prevention [1,2]. From the perspective of motor control research, in human postural control, the visual, somatosensory, and vestibular senses are crucial, as they are integrated in the central nervous system and utilized for maintaining posture [3]. Proprioception, a type of somatosensory sensation, contributes significantly to postural control [4]. Proprioception is classified into kinesthesia, joint position sense, vibration sense, and deep pain sense. Joint position sense is a static characteristic that detects the position and orientation of limb joints [5]. This sense allows the perception of the relative positions of the arms and legs [6], significantly contributing to the postural control function of proprioception [7].

Methods for evaluating lower limb proprioception include measuring joint position sense and center-of-gravity sway. Joint position sense is assessed by the ability to accurately reproduce a specific joint angle and is typically evaluated using reproduction or imitation techniques [8,9,10]. Devices such as the BIODEX system are used to objectively measure ankle and knee joint positions in a non-weight-bearing position [8]. However, this method requires large-scale experimental equipment and limits the experimental environment. Additionally, it requires specialized knowledge and skills. In contrast, measuring the center-of-gravity sway using a force plate allows testing in a weight-bearing standing position, offering practical advantages. However, this approach does not exclusively assess foot proprioception and may be influenced by visual and vestibular inputs and neuromuscular control, depending on the conditions applied [8]. If a method allows researchers to evaluate lower limb proprioception without being limited to a specific experimental environment, it would be possible for field personnel such as instructors, coaches, supervisors, and parents to perform measurements, and this may contribute to injury prevention in athletes.

A previous study used a device capable of varying the angle of the floor inclination to determine the threshold at which the participants could distinctly perceive the tilt [11]. In that study, the recognition rate for detecting floor inclination while seated on the floor or in a chair was approximately 100% at angles between 4.0° and 5.0°. However, previous study has focused on subjective assessments and did not specifically address the sensory functions related to postural control. The role of ankle proprioception was not considered, and without fixation of the torso, other senses such as vestibular, gluteal, and tactile sensations were likely involved. Fitzpatrick et al. [12] examined the threshold of perception for postural sway and reported that ankle proprioception, a type of somatosensory input, was more sensitive than the vestibular and visual inputs for detecting postural sway in a normal standing position. To date, a method for identifying floor inclination in a standing position on the feet and using it to evaluate ankle proprioception has not been employed. However, building on the approach of Uno et al. [11], it is possible to assess ankle proprioception by identifying the presence of floor tilt while standing still with both feet on the floor. Based on the findings of Fitzpatrick et al., if finer angles of floor tilt can be identified compared to previous studies [12], ankle proprioception can be utilized to sense these tilts. This study aimed to evaluate ankle proprioception by probing the tilt sensation ability of study participants using a tilt sensation measurement method and examine its reliability.

Injuries to the lower limbs are common across sports, regardless of the type of sport, owing to the nature of movement and action involved in predominantly terrestrial activities [13,14,15,16,17]. In particular, ankle sprains have high incidence and recurrence rates among sports injuries [18,19,20]. Willem et al. [21] conducted longitudinal studies on college students and identified a reduced sense of ankle joint position as a risk factor for ankle sprains. Additionally, a lowered sense of knee joint position has also been cited as a risk factor for injuries to the anterior cruciate ligament, a common type of sports injury [22]. These findings suggest that diminished proprioception in the lower limbs may lead to sports injuries.

Compared with studies focusing on sports injuries and lower limb proprioception in adult athletes, research targeting children is scarce [23]. It is believed that children between the ages of 7 and 10 integrate and utilize sensory inputs for postural control in a manner similar to adults [24]. However, there is no clear evidence concerning the development of lower-limb proprioception during childhood. Understanding the development of proprioception in the lower limbs of children during the growth phases and analyzing its relationship with sports injuries is crucial for preventing injuries in junior athletes. This study aimed to elucidate the relationship between the tilt sensation abilities of junior athletes, their physical development, and sports injuries using a newly developed tilt sensation measurement method.

## 2. Materials and Methods

### 2.1. Participants

#### 2.1.1. Experiment 1

The participants were 22 university students (10 men and 12 women). The physical characteristics of the participants are listed in Table 1.

#### 2.1.2. Experiment 2

The participants consisted of 40 individuals (19 males and 21 females) ranging from Grade-4 elementary to Grade-3 junior high school students who were actively engaged in one of the following competitive sports: badminton, fencing, climbing, canoeing, or rowing, with participants attending specialized training sessions about twice a week.

### 2.2. Ethical Approval

Prior to the experiment, participants or their family members were informed of the purpose of the experiment, the procedure, their rights, and any potential risks, and written consent for their participation was obtained. This study was approved by the Ethics Committee of the Kyoto Institute of Technology and conducted in accordance with the Declaration of Helsinki (Protocol Number 2022-14).

### 2.3. Experimental Procedure

#### 2.3.1. Experiment 1

A step platform (manufactured by Grong) was used to set up a downward, leftward tilt. In this study, we utilized a combination of boards with 2 different thicknesses, 0.6 cm and 0.9 cm, to create approximately 14 different tilt angles (0.36, 0.54, 0.72, 0.90, 1.1, 1.3, 1.6, 1.9, 2.0, 2.1, 2.3, 2.5, 2.7, and 2.9°) at intervals of approximately 0.2–0.3 degrees. Participants wore an eye mask and were instructed to stand stationary on the step platform and answer “yes” if they felt there was a tilt and “no” if they felt there was no tilt. In addition, the participants were informed of the following three points of caution regarding the operation: The first was to keep the feet floating while waiting for the tilt to be set in the chair position, to prevent the participant from remembering the sensation of 0°. The second was to get off the chair slightly more vigorously than slowly when standing on the step platform. Instead of placing the foot on the step platform, bending the knee, and then slowly standing up, the person was instructed to jump onto the step platform and immediately extend the knee to stand up. This technique prevents the sensation of the tilt angle while bending the knees on the platform in a stationary standing position. The third was to keep both feet open with a fist-width space between them while standing stationary on the step platform. Based on the above precautions, the participants were given several practice sessions on sensing the actual tilt of the step platform. However, we did not inform the participants about the correctness of their answers to prevent them from learning. During the experiment, music was played continuously over the speakers to prevent the participants from hearing sounds made during the tilt adjustment.

The ability to sense the tilt angle was determined using the following procedure: One angle of tilt among the 14 types was judged on the basis of a binomial distribution as to whether or not it could be sensed. Initially, one trial was conducted as practice, followed by nine actual trials. In total, ten trials were performed, with “tilt present” and “tilt absent” conditions presented five times each in a random sequence. The first trial, being for practice, was not considered in evaluating the correctness of participant responses. Regarding the success or failure of the tilt sensation of an angle, if the participant was able to correctly identify “with tilt” when standing on a step at a certain angle, and “without tilt” when standing horizontally at 0 degrees in all nine trials, the participant was considered to be able to identify the difference between the presence and absence of tilt, and thus to be able to sense the tilt of that angle. If an error was made in only one of the nine trials, another set of nine trials was conducted. A participant was considered capable of sensing the tilt if he or she made three or fewer errors out of 18 trials. However, if two errors were made within the first nine trials, the evaluation was terminated, and the participant was deemed incapable of sensing the tilt of that angle.

In this study, the minimum angle at which the presence or absence of a tilt can be sensed was determined by the following procedure. Initially, all participants were assessed for their ability to sense a tilt of 1.6 degrees. If they could, they were set two levels lower at 1.1 degrees. If a 1.1 degree tilt was sensed, the angle was further reduced by two levels to 0.72 degrees. If the participants failed to sense the 1.1-degree tilt, an intermediate angle of 1.3 degrees, lying between the sensible and non-sensible angles, was tested. In cases where the 0.72-degree tilt was not sensed, the angle was adjusted to 0.9 degrees for measurement. Conversely, if a 1.6 degree tilt was not sensed, the angle was increased by two levels to 2.0 degrees. If the participants failed to sense a 2.0 degree tilt, the angle was further increased to 2.3 degrees. If a 2.0 degree tilt was sensed, the angle was then set to 1.9 degrees, lying between 1.6 and 2.0 degrees. The evaluation of tilt angles proceeded in this manner, and the minimum angle at which the presence or absence of a tilt could be identified was determined as the minimum angle for tilt sensation. After the first session of measurements, participants had a rest period of at least 30 min. To assess the reliability of the measurement method, a second session of measurements was held after the rest period.

#### 2.3.2. Experiment 2

Similar to Experiment 1, a step platform was used to create a leftward declining tilt. In Experiment 2, we combined 0.6 cm and 0.9 cm plates with six different tilt angles (0.54, 1.1, 1.6, 2.1, 2.7, and 3.2°) in order to shorten the experimental time and reduce the burden on the participant and experimenter. The methods and precautions for identifying tilt remained the same as in Experiment 1. One angle of tilt among the six types was judged on the basis of a binomial distribution as to whether or not it could be sensed.

Initially, one trial was conducted as practice, followed by seven trials. In total, eight trials were performed, with “tilt present” and “tilt absent” conditions presented four times each in a random sequence. Since the first trial was for practice, the correctness of the participant responses in that trial was not used as a criterion for judgment.

Regarding the success or failure of the sensation of tilt, if the participant was able to correctly identify “with tilt” when standing on a step at a certain angle, and “without tilt” when standing horizontally at 0 degrees in all seven trials, the participant was considered to be able to identify the difference between the presence and absence of tilt, and thus to be able to sense the tilt of that angle. If an error was made in only one of the seven trials, another set of seven trials was conducted. A participant was considered capable of sensing the tilt if he or she made two or fewer errors out of fourteen trials. However, if two errors were made within the first seven trials, the evaluation was terminated at that point, and the participant was deemed incapable of sensing the tilt of that angle.

As in Experiment 1, the minimum angle at which the presence or absence of tilt could be identified was selected using the up-and-down method. Initially, all participants were assessed for their ability to sense a tilt of 3.2 degrees. If successful, the angle was decreased by two levels to 2.1 degrees. If a tilt of 3.2 degrees was not sensed, the test was terminated. If a 2.1 degree tilt was sensed, the angle was further reduced by two levels to 1.1 degrees. If a 2.1 degree tilt could not be sensed, the participant was tested at 2.7 degrees between the sensed and unsensed angles, i.e., between 3.2 and 2.1 degrees. If a 1.1 degree tilt was sensed, the angle was further reduced to 0.54 degrees. If participants failed to sense the 1.1 degree tilt, an intermediate angle of 1.6 degrees, lying between 2.1 and 1.1 degrees, was tested.

### 2.4. Medical Checks

#### Experiment 2

In the medical check, the presence or absence of knee injuries and lower leg and foot injuries was verified, based on diagnoses by three orthopedic surgeons and the results of subjective symptoms and physical examination findings, including tenderness tests. For the knee, athletes were considered positive for knee injuries if diagnosed with any of the following conditions: Osgood-Schlatter disease, jumper’s knee, or meniscal injuries. As for lower leg and foot injuries, the assessment included shin splints, flat feet, painful os peroneum syndrome, Achilles tendonitis, and Sever’s disease, with athletes diagnosed with any of these conditions being classified as positive for lower leg and foot injuries. In addition, athletes with either knee or lower leg and foot injuries were also classified as having lower limb injuries.

### 2.5. Statistical Analysis

#### 2.5.1. Experiment 1

We performed the Wilcoxon test to examine differences between the first and second sessions in the minimum angle for tilt sensation. To examine reliability, Spearman’s rank correlation coefficients were determined for the first and second sessions at the minimum angle for tilt sensation. To examine the relationship between the minimum angle for tilt sensation and body size, Spearman’s rank correlation coefficient was calculated. To investigate the differences in the minimum angle for tilt sensation by sex, the Mann-Whitney U test was conducted. All statistical analyses were performed using SPSS version 27 software (IBM Corp, Armonk, NY, USA). Statistical significance was set at *p* < 0.05.

#### 2.5.2. Experiment 2

To explore the relationship between the minimal angle for tilt sensation, body size, and age, the Spearman’s rank correlation coefficient was calculated. The Mann-Whitney U test was used to investigate sex differences in the minimal angle for tilt sensation. Based on the presence or absence of tilt sensation at 1.1, 1.6, and 2.1 degrees, participants were categorized into sensed and unsensed groups. The proportions of sensed groups among elementary and junior high school students at these angles were examined using the chi-square test. A chi-square test was used to examine the relationship between the presence or absence of tilt sensation and lower limb injuries at these angles. Additionally, to determine if there were differences in the minimal angle of tilt sensation among elementary, junior high school, and university students (including university students in Experiment 1), angles were ranked from 1 to 6 based on the six angles used in Experiment 2, starting at 0.54 degrees. The failure to sense a 3.2 degree tilt was classified as 7. The Kruskal-Wallis test, followed by Dunn-Bonferroni post hoc tests, was conducted. All statistical analyses were performed using SPSS version 27 software (IBM Corp). Statistical significance was set at *p* < 0.05.

## 3. Results

### 3.1. Experiment 1

**Figure 1:**
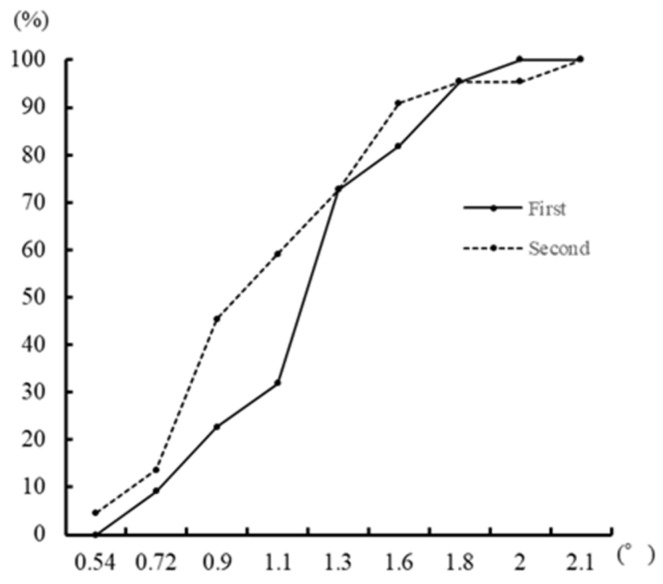
Cumulative frequencies at first and second session.

The percentage of cumulative degrees of the minimum angle of tilt sensation in the first and second sessions is shown in Figure 1. To examine the difference between the first and second sessions, a Wilcoxson’s test was performed, and no significant difference was found (*Z* = −1.594, *p* = 0.111). For assessing the reliability of measurements, the Spearman correlation coefficient was calculated, revealing a moderate and significant correlation between the sessions (*r* = 0.504, *p* = 0.017). Additionally, we examined the relationship between the minimum angle for tilt sensation and body size characteristics using Spearman’s rank correlation. No significant correlations were observed with height (*r* = 0.017, *p* = 0.940) or weight (*r* = 0.214, *p* = 0.339). Moreover, the Mann-Whitney U test was utilized to evaluate differences in the minimum angle of tilt sensation by sex, and showed no significant differences (*U* = 60.0, *p* = 1.000).

### 3.2. Experiment 2

Spearman’s rank correlation analysis revealed a significant negative correlation between age in years and the minimum angle for tilt sensation (*r* = −0.414, *p* = 0.008). No significant differences were found between the sexes in the minimal angle for tilt sensation when tested using the Mann-Whitney U test (*U* = 196.5, *p* = 0.933). Our data revealed a relationship between age and the minimal angle for tilt sensation. Therefore, the relationship between body size and the minimal angle for tilt sensation was analyzed separately for elementary and junior high school students (Table 2). There was no significant relationship between body size and the tilt sensation angle for both elementary and junior high school students. The number and percentage of elementary and junior high school students in the sensed group at each angle are detailed in Table 3. The proportion of junior high school students was significantly higher than that of elementary students in both the 1.1 degree and 1.6 degree sensed groups (1.1 degree sensed group; *χ*^2^ = 8.839, *p* = 0.003. 1.6 degree sensed group; *χ*^2^ = 4.038, *p* = 0.044). There was no significant difference between elementary and junior high school students in the 2.1 degree sensed group (*χ*^2^ = 2.338, *p* = 0.126).

Additionally, when examining the minimally sensitive angle classified by rank among elementary, junior high school, and university students, including the university students from Experiment 1, a significant difference was observed among the three groups (*χ*^2^ = 12.255, *p* = 0.002). Multiple comparisons showed that elementary school students had significantly lower sensation ranks compared to junior high school students (*p* = 0.002). No significant differences in ranks were observed between elementary school and university students, or between junior high school and university students (elementary school vs. university; *p* = 0.163; junior high school vs. university; *p* = 0.278).

Table 4 shows the positive rates of injuries in sensed and unsensed groups of elementary and junior high school students at tilt angles of 1.1 degrees, 1.6 degrees, and 2.1 degrees. For elementary school students, no significant differences in positive injury rates were observed between the sensed and unsensed groups at any angle for lower limb injuries, knee injuries, and lower leg and foot injuries. In contrast, for junior high school students, there was no significant difference between the sensed and unsensed groups for lower limb, lower leg, and foot injuries, however, for knee injuries, the unsensed group had significantly higher positive rates at 1.6 degrees and 2.1 degrees (1.6 degrees; *χ*^2^ = 4.622, *p* = 0.032, 2.1°; *χ*^2^ = 4.622, *p* = 0.032).

## 4. Discussion

In this study, we attempted to assess ankle proprioception by using a measurement method to identify whether the floor surface is tilted while standing on both feet. The results of Experiment 1 showed that the maximal angle of tilt sensation for all participants was 2.1°, indicating that the participants were able to sense a tilt of 2.1° or less. Previous studies conducted in flat-sitting or chair-seated positions that probably utilized vestibular sensation and proprioception of the gluteal region have reported recognition thresholds between 4°and 5° [11]. Therefore, the results of the present study demonstrate the ability to identify smaller angles compared with earlier research [11]. Fitzpatrick et al. [12], in their experimentally controlled physiological study, evaluated the perception thresholds of sensory organs to stimuli, individually assessing vestibular sensation, visual, and ankle joint proprioception. They noted that the proprioception of the ankle joint was more acute than the vestibular sensory inputs in normal standing positions. Therefore, we believe that the method employed in the present study effectively evaluated ankle proprioception. Additionally, the reliability of the measurement method was assessed by examining the differences in the minimal angles between the first and second sessions; no significant differences were found, and a moderate correlation (*r* = 0.504) was observed between the minimal angles for tilt sensation in the first and second sessions. Therefore, the method used in this study is considered valid for assessing ankle proprioception.

In Experiment 2, the sensation rates for tilts of 1.1° and 1.6° exhibited age-related differences; junior high school students demonstrated higher tilt sensation abilities than elementary school students. Additionally, when university students from Experiment 1 were included in the analysis, junior high school students exhibited values comparable to those of university students. Previous research on postural sway from the ages of 4 to 17 years has reported that sway in the anterior-posterior and lateral directions decreases linearly until the age of 9 years, followed by a gradual decline as age increases [25]. The results of the present study also showed a developmental trend similar to those of previous studies, confirming that ankle proprioception improved during the transition from elementary to junior high school. The measurement method used in this study evaluates the proprioception of the ankle, and it is known that proprioception also contributes to the ability to control posture [3,4]. Therefore, improvements in abilities observed during the transitional period from elementary to junior high school, as demonstrated in this study and in previous studies, suggest that enhancements in proprioception may contribute to increased postural stability. Furthermore, in the tilt sensation assessment conducted in this study, the elementary school students sensed tilts of 1.1° and 1.6° at rates of 33.3% and 66.6%, respectively. Although some elementary school students exhibited a proprioception ability comparable to junior high school and university students, the tilt sensation rates were lower compared to junior high school students. These findings suggest that there is individual variability in the development of proprioception during the elementary school period.

In this study, physical factors such as height and weight did not influence tilt sensation abilities. Previous studies investigating the relationship between static standing balance and body size in adults have reported that taller individuals and those with greater body weight tend to experience more significant sway in their center of gravity [26,27]. This suggested that, in the case of this study, body sway due to differences in body size may affect the ability to identify the presence or absence of tilt; however, no such association was found. In our present study, the participants adopted a method of standing that involved landing on both feet with some momentum on a step platform, and this may have minimized the sway caused by differences in height and weight during standing.

In junior high school students, the failure to sense tilts of 1.6° and 2.1° tended to increase the positive rate of knee injuries. Previous longitudinal studies examining the risk factors for lower limb injuries identified a decrease in the sense of ankle joint position as a risk factor for ankle sprains and a decrease in sense of the knee position as a risk factor for anterior cruciate ligament injuries [21,22,28]. Thus, reduced proprioception is considered a risk factor for lower-limb injuries. Therefore, the lack of tilt sensation ability was suggested to be linked to a decreased sense of ankle joint position, potentially leading to knee injuries. However, unlike previous studies, this study revealed that the relationship between ankle proprioception and injury lies not at the foot, but at the knee. The control of the center of gravity of the body during landing actions is thought to be conducted through the kinetic chain of the lower limbs, including the ankles, knees, and hips, as well as through overall body coordination [29]. Therefore, if ankle proprioception is low, the body’s movements and posture adjustments due to stretch reflexes may not occur during awkward landings, potentially causing mechanical stress on the knee. Additionally, compensatory movements may occur during repetitive actions, such as landing, affecting different parts of the body other than the feet. Previous research has reported that training to improve proprioception can enhance proprioceptive function or reduce the incidence of lower-limb injuries [30,31,32,33,34]. Consequently, measuring tilt sensation ability using the method devised in this study and implementing proprioception improvement training if the ability is low can be considered an effective means of injury prevention. Additionally, for individuals with low tilt sensation abilities, meticulous conditioning of the knees and seeking medical attention if discomfort is present can facilitate the early detection of injuries.

In elementary school students, no association was found between lower limb injuries and tilt sensation abilities. It is known that there are individual differences in growth and development during childhood [35,36,37], and this study demonstrated that elementary school students showed greater individual variability in proprioception in the ankle compared to junior high school students. Therefore, using the tilt sensation measurement method developed in this study as a tool for injury prevention and early detection may be more effective in evaluating junior high school or older students, in whom the impact of variability in proprioceptive development is less significant. Additionally, from the perspective of the sample size, the possibility that significant differences were not detected cannot be ruled out.

In junior high school students, our results indicated an association between tilt sensation abilities and knee injuries, suggesting that knee injuries may also result in reduced tilt sensation abilities. Therefore, if the method of measurement used in this study indicates a low tilt sensation ability and even slight discomfort in the knee, it is suggested that seeking medical attention could help detect and prevent the progression of the injuries. These findings regarding the relationship between proprioception and knee injuries may not only apply to the athletic discipline examined in this study but also to other disciplines that involve the lower limbs, such as running.

This study has two major limitations in terms of the research design and study participants. The causal relationship between knee injuries and injuries to the lower legs and feet could not be elucidated for the ankle because this study adopted a cross-sectional study design. Specifically, it cannot be determined whether low-tilt sensation leads to injuries, or whether injuries lead to low-tilt sensation. To determine whether poor tilt sensation leads to injuries or if injuries result in decreased tilt sensation abilities, future longitudinal studies using both prospective and retrospective study designs are needed. These studies should determine the relationship between tilt sensation test results and subsequent medical checks to identify the factors that contribute to sports injuries in growing children. Although our present study included participants from elementary school through university, extending the study to infants and the elderly may require additional modifications, such as lowering the step platform to prevent falls.

## 5. Conclusions

In this study, we attempted to evaluate ankle proprioception using a method that involved standing on both feet and identifying the presence and absence of tilt with reference to the floor surface. These results indicate that the method developed in this study effectively evaluates ankle proprioception and can be considered a reliable measurement method for assessing tilt sensation. Age-related differences were observed in ankle proprioception, with junior high school students demonstrating higher tilt sensation abilities than elementary school students. Additionally, it was suggested that there is individual variability in the development of proprioception among elementary school students. Furthermore, a relationship between low tilt sensation abilities and knee injuries in junior high school students has been suggested. Using the tilt sensation assessment test devised in this study to measure tilt sensation abilities in junior high school students and evaluate foot proprioception could play a crucial role in the prevention and early detection of injuries. Furthermore, preventing injuries may lead to higher performance and may be useful in recruiting players.

## Figures and Tables

**Table 1:** Physical characteristics of university students (*n* = 22).

	Men	Women	Overall
Age (years)	20.3 ± 0.9	20.8 ± 0.9	20.6 ± 0.9
Height (cm)	171.2 ± 4.4	157.8 ± 5.2	163.9 ± 8.3
Weight (kg)	64.2 ± 10.6	51.5 ± 5.9	57.3 ± 10.4

Values are mean ± standard deviation.

**Table 2:** Relationship between body size and tilt sensitive minimum angle in elementary and junior high school students.

	Elementary School Student				Junior High School Student			
	M ± SD	*n*	Spearman’s rho	*p*	M ± SD	*n*	Spearman’s rho	*p*
Height (cm)	141.4 ± 7.6	22	−0.162	0.472	162.2 ± 6.7	16	−0.024	0.930
Weight (kg)	33.4 ± 6.1	19	0.184	0.450	48.8 ± 7.0	16	0.169	0.532

M, mean; SD, standard deviation.

**Table 3:** Proportion of the sensed groups by elementary and junior high school categories.

	Elementary School Student (*n* = 24)		Junior High School Student (*n* = 16)		*χ* ^2^	*p*
	*n*	%	*n*	%		
1.1°	8	33.3	13	81.3	8.839	0.003 **
1.6°	16	66.7	15	93.8	4.038	0.044 *
2.1°	18	75.0	15	93.8	2.338	0.126

*, *p* < 0.05; **, *p* < 0.01.

**Table 4:** Proportion of injuries occurring in the sensed and unsensed groups.

	Elementary School Student				Junior High School Student			
	Sensed Group	Unsensed Group	*χ* ^2^	*p*	Sensed Group	Unsensed Group	*χ* ^2^	*p*
Lower limbs								
1.1°	2 (25.0)	7 (43.8)	0.800	0.371	8 (61.5)	1 (33.3)	0.788	0.375
1.6°	4 (25.0)	5 (62.5)	3.200	0.074	8 (53.3)	1 (100.0)	0.830	0.362
2.1°	5 (27.8)	4 (66.7)	2.904	0.088	8 (53.3)	1 (100.0)	0.830	0.362
Knee								
1.1°	2 (25.0)	2 (12.5)	0.600	0.439	2 (15.4)	1 (33.3)	0.515	0.473
1.6°	2 (12.5)	2 (25.0)	0.600	0.439	2 (13.3)	1 (100.0)	4.622	0.032 *
2.1°	3 (16.7)	1 (16.7)	0.000	1.000	2 (13.3)	1 (100.0)	4.622	0.032 *
Lower leg and foot								
1.1°	2 (25.0)	6 (37.5)	0.375	0.540	7 (53.8)	1 (33.3)	0.410	0.522
1.6°	4 (25.0)	4 (50.0)	1.500	0.211	7 (46.7)	1 (100.0)	1.067	0.302
2.1°	5 (27.8)	3 (50.0)	1.000	0.317	7 (46.7)	1 (100.0)	1.067	0.302

*, *p* < 0.05

## Data Availability

All experimental data files are available from the figshare database. https://doi.org/10.6084/m9.figshare.25928779.v1 (accessed on 30 May 2024).
